# Combining short stent implantation and drug-eluting stenting for routine use yields a low restenosis rate

**DOI:** 10.1186/1468-6708-6-18

**Published:** 2005-12-13

**Authors:** Ulrich Dietz, Cheryl Dauer, Heinz Lambertz

**Affiliations:** 1 German Clinic for Diagnostic, Wiesbaden, Germany

## Abstract

**Background:**

Stent length serves as a predictor of restenosis in use of bare metal stents (BMS). This has been demonstrated in a feasibility study that used a single short BMS implant (<9 mm) in a high proportion of lesions; the study observed a low rate of restenosis.

**Methods:**

We performed a pilot prospective study to investigate in a series of consecutive patients the immediate and long-term effects of implantation of either 1) a single short BMS for all lesions with low probability of restenosis or 2) a drug-eluting stent (DES) for all other lesions.

**Results:**

The 200 patients studied had 236 coronary artery lesions that were treated with short BMS in 168/236 patients (71.2%) and with DES in 68/236 patients (28.8%). Angiographic success was achieved in 230/236 lesions (97.5%) and procedural success in 194/200 patients (97.0%). Restenosis occurred in 15/153 lesions (9.8%) after short BMS, in 3/62 lesions (4.8%) after DES, and in 18/215 of all lesions (8.4%) angiographically controlled after six to eight months. Target vessel revascularization was performed in 16/218 lesion (7.4%).

**Conclusion:**

Most of the coronary artery lesions in this small group of consecutive patients were treated sufficiently with a single BMS implant. This differential approach of treating suitable lesions in medium- to large-sized vessels with a single short BMS device and treating all other lesions with a DES implant resulted in a low incidence of restenosis.

## Introduction

Sirolimus- and Tarcolimus-eluting stents reduce restenosis rates in larger vessels and in longer lesions as compared to bare metal stents (BMS) [1,2]. The low restenosis rates found with these drug-eluting stents (DES) suggests that all lesions could benefit from this type of stent. One concern about their use in the majority of lesions relates to cost, since these stents are much more expensive than bare metal stents [2]. A recently published cost-effectiveness analysis showed that treatment with DES would impart cost savings in patients with a BMS target vessel revascularization (TVR) rate >20% and cost effectiveness in patients with a BMS TVR rate >12% [3]. Stent length and vessel size represent primary factors affecting BMS restenosis rates [4]. Moreover, implantation of a BMS that is longer than the lesion itself increases the risk of restenosis independently [5].

In a previous prospective study, we demonstrated that stenting of only the most significant obstruction of a lesion after balloon dilatation or use of direct stenting alone was feasible in a high proportion of cases yielding low rates of stenosis. [6]. In another prospective study, we observed that short stenting significantly reduced the restenosis rate as compared to conventional stenting, which involves placement of the stent over the entire stenotic segment of a vessel [7]. In this latter study, vessel size only was found to predict restenosis after short stenting. In the present pilot study, we report on our ongoing evaluations by comparing a) short stenting with BMS in lesions with low probability of restenosis to b) stenting with DES in lesions with predicted restenosis rates of >10% after BMS.

## Methods

### Patients

Two hundred consecutive patients with symptoms of angina pectoris, a positive exercise tolerance test, or both, and coronary arteries with >60% diameter stenoses were included in this study. Patients with lesions in the left main artery and patients treated for myocardial infarction were excluded.

### Procedures

Clopidogrel 75 mg was administered daily starting 4 days prior to elective interventions. All patients received acetylsalicylic acid for at least 14 days prior to intervention and heparin (5000 IU) as an intravenous bolus before the procedure and then by infusion during the procedure, at dose levels targeting a 2.5-fold prolongation of activated prothrombin time (aPTT). Percutaneous transluminal coronary angioplasty was performed initially in most cases using semi-compliant balloons. The balloon length was 15 mm for short lesions (<10 mm) and 20 mm for longer lesions. Balloon sizes were selected to achieve a balloon/artery ratio of 1.0 to 1.2 when fully expanded at >10 atm.

#### Use of BMS stents

A short BMS (<10 mm) was considered for vessels > 2.7 mm, to be implanted either:

1. By direct stenting if the lesion had a length <10 mm located in the proximal part of the left anterior descending coronary artery, or of the right coronary artery; or

2. After balloon dilatation of a longer lesion if angiography performed 5 min after the initial intervention showed a residual stenosis >30% that reflected a lesion ≤ 10 mm in length, in the absence of a longer dissection.

#### Use of DES stents

For all other lesions not suitable for a short BMS, a DES was implanted.

All stents, premounted on semi-compliant balloons, were sized to achieve a stent to artery ratio of 1.1 at >12 atm. The short BMS included 168 MultiLink stents, while the DES involved 53 Cypher stents and 15 Taxus stents. After the procedure, all patients were prescribed acetylsalicyclic acid 100 mg daily indefinitely. For patients who had received a stent, clopidogrel 75 mg daily was also prescribed for 4 weeks. Patients with hypercholesterolemia were strongly advised to take a cholesterol synthesis inhibitor regularly. Six months and eight months after implantation of the BMS and DES implants, respectively, control angiography was performed using projections that were identical to those employed during the intervention.

### Quantitative coronary angiography

The CAAS II Research System (Pie Medical Imaging, Maastricht, The Netherlands) was used for automated contour detection and quantification. The system and validation data are described elsewhere [8]. The measurement procedure was previously published in detail [9]. Angiography was performed before and after all interventions and at angiographic follow-up using identical projections and analyses. Frames were selected as recommended by Herrington and Walford [10]. Analyses followed the guidelines proposed by Reiber *et al*. [11]. Lesion length was the distance along a vessel segment characterized by a diameter stenosis >50%, as compared to the reference segment. Lesion length, mean diameter within the lesion (mean stenosis diameter), and minimum lumen diameter (MLD) were calculated for the target vessel segment. In addition, mean diameter stenosis and minimum lumen diameter were measured at a distance of 5 mm from each stent edge. Restenosis was defined as > 50% in segment diameter stenosis at angiographic follow-up.

### Statistical analyses

Continuous data are expressed as the mean ± the standard deviation. Chi-square tests were used to compare categorical variables, and the Mann-Whitney test was employed to compare continuous variables. Statistical significance was accepted at *p*<0.05.

## Results

The study included 200 consecutive patients with a total of 236 treated lesions (mean 1.2 lesions/patient). Baseline clinical and angiographic characteristics are shown in Tables 1 and 2. Balloon dilatation was performed initially in 198/236 (83.9%) coronary artery lesions, and direct stenting with a short BMS was performed in 38/236 (16.1%). A short BMS after balloon angioplasty was judged suitable in 130/236 (55.1%) lesions, and a DES was implanted in 68/236 (28.8%) lesions that were deemed to be unsuitable for BMS. An additional stent was implanted subsequent to the first procedure in cases involving persistent residual stenosis >30% or a dissection in nine cases (5.4%). Three DES implants could not be deployed; the results of the balloon angioplasty were not further manipulated.

**Table 1:** Baseline characteristics

Variable	All patients N = 200
Age (years)	62 ± 10
Males	154 (77.0%)
CCS III – IV	78 (38.0%)
Hypertension	153 (76.5%)
Hypercholesterinemia	155 (77.5%)
Diabetes mellitus	48 (24.0%)
Multivessel coronary disease	97 (48.5%)

± : Standard deviation.

CCS: Canadian Cardiovascular Society classification.

**Table 2:** Lesion baseline characteristics

Variable	All lesions N = 236
De novo lesion	227 (94.9%)
Total occlusion	8 (3.4%)
Left anterior descending artery	83 (35.2%)
Left circumflex artery	53 (22.5%)
Right coronary artery	73 (30.9%)
Coronary artery side branches	23 (9.7%)
Venous coronary bypass	4 (1.7%)
Lesion morphology	
Eccentric	157 (66.5%)
Diffuse	33 (14.0%)
AHA type A/B1	93 (39.4%)
AHA type B2/C	143 (60.6%)

AHA: American Heart Association.

As compared to BMS implantation, DES implants were used more frequently for side branch lesions (*p *= 0.03) and longer lesions (*p*<0.0001) in smaller vessels (*p *= 0.0001). The DES devices were significantly smaller (2.7 ± 0.2 mm vs. 3.0 ± 0.4 mm, p < 0.0001) and longer (18.4 ± 5 mm vs. 8.3 ± 0.2 mm, p < 0.0001) than the short BMS implants (Table 3).

**Table 3:** Procedural data and baseline characteristics of quantitative coronary angiography

Variable	Short BMS n = 168	DES n = 68	*p*	All Stents n = 236
Stent length (mm)	8.3 ± 0.2	18.4 ± 5	<0.0001	11.2 ± 8
Nominal device diameter (mm)	3.0 ± 0.4	2.7 ± 0.2	<0.0001	2.9 ± 0.5
Inflation pressure (atm)	13 ± 3	13 ± 3	ns	13 ± 3
Device/artery ratio	1.12 ± 0.1	1.14 ± 0.1	ns	1.13 ± 0.1
Reference diameter (mm)	3.1 ± 0.4	2.8 ± 0.3	<0.0001	3.0 ± 0.5
Diameter stenosis before (%)	74 ± 14	76 ± 13	<0.0001	75 ± 14
MLD before (mm)	0.77 ± 0.4	0.73 ± 0.4	ns	0.76 ± 0.3
Plaque area (mm^2^)	9 ± 5	14 ± 7	0.0001	10 ± 6
Lesion length (mm)	8.7 ± 3	14.6 ± 5	<0.0001	9.8 ± 5

Nominal device diameter: Stent diameter at nominal inflation pressure.

± : Standard deviation.

ns: non-significant.

MLD: Mean lumen diameter.

Angiographic analysis revealed that residual plaque outside the short BMS contributed to 25% to 45% residual diameter stenosis in 15/168 (8.9%) lesions, and none after DES implantation.

### Immediate results

Angiographic success (post-intervention diameter <30% within the stent) was the same for short BMS and DES (164/168; 97.6%, and 66/68; 97.0%) as was procedural success (angiographic success and no MACE [myocardial infarction, reintervention, death]), which was accomplished in 194 /200 (97.0%) patients (Table 4). Major cardiac events (myocardial infarction, repeat target vessel revascularization or death) occurred in two patients.

**Table 4:** Clinical and angiographic outcomes

Variable	All lesions n = 236
Angiographic success	230 (97.5%)
Procedural success	194/200 (97.0%)
Final minimum lumen diameter (mm)	2.9 ± 0.3
Final diameter stenosis (%)	15 ± 12
Acute gain (mm)	2.2 ± 0.3
Final plaque area (mm^2^)	2.8 ± 2.2

### Clinical follow-up

Angina pectoris (Canadian Cardiovascular Society classes III or IV) recurred during follow-up in 3 patients, and major cardiac events were experienced by 3 patients, of whom 2 died during the 8-month follow-up period while the third underwent elective coronary artery bypass grafting. Target vessel revascularization was performed in 16/218 (7.4%) lesions at follow-up.

### Angiographic follow-up

In 180/192 eligible patients, angiography was repeated at 193 ± 25 days on 215/226 (95.1%) lesions (Table 5). Restenosis occurred in 18/215 (8.4%) of all lesions, in 15/153 (9.8%) lesions treated with a short BMS and in 3/62 (4.8%) lesions after DES. According to univariate analysis, the restenosis rate was higher when an additional stent had been implanted after short BMS. No other clinical or angiographic parameter was associated with increased risk of restenosis.

**Table 5:** Angiographic follow-up

Variable	All lesions n = 215
Percent diameter stenosis	33 ± 20
Minimum lumen diameter (mm)	2.3 ± 0.6
Late loss (mm)	0.58 ± 0.5
Loss index	26 ± 15
Net gain (mm)	1.5 ± 0.7
Net gain index	51 ± 24
Neo-plaque area (mm^2^)	1.8 ± 3.5
Net plaque reduction (mm^2^)	6.2 ± 4
Restenosis (%)	18 (8.4%)

Net gain index: Net gain / vessel size × 100.

Neo-plaque area: Plaque area at follow-up minus final plaque area.

Net plaque reduction: plaque area before minus plaque area at follow-up.

Loss index: Late loss / acute gain * 100

± : Standard deviation.

## Discussion

Although BMS implants in many lesions have reduced the restenosis rate as compared to PTCA, stent implantation in the clinical setting continues to yield restenosis rates exceeding 20% [12]. In general, in-stent restenosis (ISR) can be predicted based on stent size and stent length and based on the presence of diabetes mellitus [4,13]. Previous analyses have demonstrated that stent length can serve as a predictor of ISR independent of lesion length [14,15]. A recent meta-analysis of intervention trials noted that stent length exceeds lesion length in about 90% of interventions; this excess in stent length increased the risk of ISR independent of other parameters in bare metal stenting [5].

In a previous study investigating the feasibility of routinely shortening stent length to cover only those vessel segments with significant obturating plaque and leaving nonobstructing plaque material unstented, we found it suitable to use a single stent <10 mm in 60% of all stent implantation procedures [6]. The 6-month angiographic restenosis rate in this study was 16% for all stented lesions. In a prospective study of 400 consecutive patients who underwent short stenting of their coronary artery lesions, we compared immediate and long-term results with a matched-pair control group of patients treated earlier with conventional-length stents. We found a significantly lower restenosis rate after short stenting, with no difference in the procedural success rates [7]. Vessel size was the only predictor of ISR after short stenting. In 91% of all lesions stented, the TVR rate was <10% in all vessels >2.7 mm.

With the routine use of drug-eluting stents, a lower restenosis rate can be anticipated. However, the costs of routine use of DES implants are high. A recent cost-effectiveness analysis for DES showed that these devices are cost effective when the TVR rate of BMS exceeds 12% [3].

The present pilot study was designed to demonstrate the feasibility of using short stenting in lesions with a < 10% probability of restenosis and implanting DES in all other lesions. In keeping with the results of the former investigation, a single short BMS was sufficient in the majority of interventions (252/310, 81.3%). There was a nearly 1:1 match of the stent and the lesion length. The resulting restenosis rate was within the range found in larger trials for the given stent dimensions used [13,16].

Our protocol included percutaneous transluminal coronary angioplasty as the initial procedure for most lesions. Balloon dilatation alone achieved a low residual stenosis in only a few lesions; however, it did reduce the length of obturating plaques responsible for diameter stenoses >30%. This shortening of the significant portions of the lesions contributed to the high number of lesions that could be treated adequately with single short stents. In 5.4% of these lesions, however, their length was underestimated or a stent was misplaced, thereby requiring an additional stent, which contributed to restenosis found in the short BMS group. Plaques that were adjacent to a BMS and that caused luminal narrowing < 45% exhibited no increased frequency of restenosis. This finding is consistent with previous investigations, in which incomplete plaque coverage was not associated with increased risk for restenosis.

In conjunction with the strategy of incomplete plaque coverage, Colombo *et al*. introduced the concept of 'spot stenting' long lesions (>15 mm). These researchers compared a) complete coverage of a treated long lesion to b) stenting only those stenosis segments that did not meet pre-defined intravascular ultrasound criteria for successful treatment after initial balloon dilatation [17]. They observed a lower restenosis rate in the latter group. Indeed, restricting stent surface to the minimum necessary area might reduce proliferative responses of the arterial wall to the stent [18,19].

Our findings regarding DES are comparable to those from a larger investigation of more complex lesions [1]. The overall restenosis rate of 8.4% and the TVR rate of 7.4% noted with the differential use of short bare metal stenting and DES implantation raises the question of use of DES implantation in all lesions, in terms of economic considerations. Moreover, none of the observed restenoses were diffuse or proliferative after single short BMS, a finding that is compatible with a high long-term success rate after reintervention [20].

### Methodological aspects

An exact determination of lesion length was crucial to the interpretation of angiographic results. The criterion used to measure lesion length by quantitative angiography (length of plaque causing >50% diameter reduction) reflects more precisely the extent of clinically relevant stenoses and makes it possible to standardize measurements, in contrast to overall lesion length as used by many automated contour detecting systems. However, lesion length as measured by the criterion described above is usually shorter, as compared to measuring total lesion length. Since the present study was not designed to compare immediate and long-term results of short BMS and DES, we did not perform statistical comparative analysis.

### Limitations

Our criteria for selecting a short BMS implant versus a DES implant for study participants were based on results from a non-randomized study performed earlier at our institution [7]. Selection of the stent device was based on operators' visual interpretation of vessel dimensions. This may, in a few cases, have led to misinterpretation of the actual reference diameter, as demonstrated by quantitative coronary angiography. In these cases, the other stent device was, therefore, implanted. Finally, the study was not randomized, since we intended to prove feasibility of the differential approach in a pilot study. A randomized multicenter trial now seems suitable to elucidate compatibility and cost-effectiveness of this approach with routine DES implantation.

## Conclusion

We demonstrated that: 1) for most lesions, a single short BMS is sufficient to treat the significant part of the stenotic lesion, and 2) the resulting restenosis rate using the single short BMS is low if vessels > 2.7 mm are treated. Use of DES for all other lesions that could not be treated sufficiently with a short BMS also resulted in a low restenosis rate. These findings suggest a positive synergism between short bare metal stenting and drug-eluting stenting, in terms of reducing costs while achieving overall low restenosis rates in unselected lesions.

## Competing interests

The author(s) declare that they have no competing interests.
